# Increased oral sodium chloride intake in humans amplifies selectively postprandial GLP‐1 but not GIP, CCK, and gastrin in plasma

**DOI:** 10.14814/phy2.14519

**Published:** 2020-08-07

**Authors:** Ali Asmar, Per K. Cramon, Meena Asmar, Lene Simonsen, Charlotte M. Sorensen, Sten Madsbad, Cedric Moro, Bolette Hartmann, Jens F. Rehfeld, Jens J. Holst, Peter Hovind, Boye L. Jensen, Jens Bülow

**Affiliations:** ^1^ Department of Clinical Physiology, Nuclear Medicine and PET, Rigshospitalet University of Copenhagen Copenhagen Denmark; ^2^ Department of Clinical Physiology and Nuclear Medicine Bispebjerg and Frederiksberg Hospital University Hospital of Copenhagen Copenhagen Denmark; ^3^ Department of Endocrinology Odense University Hospital Odense Denmark; ^4^ Department of Biomedical Sciences University of Copenhagen Copenhagen Denmark; ^5^ Department of Endocrinology Hvidovre Hospital University Hospital of Copenhagen Copenhagen Denmark; ^6^ Institut National de la Santé et de la Recherche Médicale (Inserm) UMR 1048 Institute of Metabolic and Cardiovascular Diseases Paul Sabatier University Toulouse France; ^7^ Novo Nordisk Foundation Center for Basic Metabolic Research University of Copenhagen Copenhagen Denmark; ^8^ Department of Clinical Biochemistry, Rigshospitalet University of Copenhagen Copenhagen Denmark; ^9^ Department of Cardiovascular and Renal Research University of Southern Denmark Odense Denmark

**Keywords:** Glucagon‐like peptide‐1, gut, gut‐kidney axis, kidney, the renin‐angiotensin‐aldosterone system

## Abstract

Human studies have demonstrated that physiologically relevant changes in circulating glucagon‐like peptide‐1 (GLP‐1) elicit a rapid increase in renal sodium excretion when combined with expansion of the extracellular fluid volume. Other studies support the involvement of various gastrointestinal hormones, e.g., gastrin and cholecystokinin (CCK) in a gut‐kidney axis, responsible for a rapid‐acting feed‐forward natriuretic mechanism. This study was designed to investigate the hypothesis that the postprandial GLP‐1 plasma concentration is sensitive to the sodium content in the meal. Under fixed sodium intake for 4 days prior to each experimental day, 10 lean healthy male participants were examined twice in random order after a 12‐hr fasting period. Arterial blood samples were collected at 10–20‐min intervals for 140 min after 75 grams of oral glucose + 6 grams of oral sodium chloride (NaCl) load versus 75 grams of glucose alone. Twenty‐four‐hour baseline urinary sodium excretions were similar between study days. Arterial GLP‐1 levels increased during both oral glucose loads and were significantly higher at the 40–80 min period during glucose + NaCl compared to glucose alone. The postprandial arterial responses of CCK, gastrin, and glucose‐dependent insulinotropic polypeptide as well as glucose, insulin, and C‐peptide did not differ between the two study days. Arterial renin, aldosterone, and natriuretic peptides levels did not change within subjects or between study days. Angiotensin II levels were significantly lower at the time GLP‐1 was higher (60–80 min) during glucose + NaCl. Sodium intake in addition to a glucose load selectively amplifies the postprandial GLP‐1 plasma concentration. Thus, GLP‐1 may be part of an acute feed‐forward mechanism for natriuresis.

## Graphical abstract

This study demonstrates that postprandial glucose‐driven glucagon‐like peptide‐1 (GLP‐1) secretion is sensitive to dietary sodium chloride intake and that this is associated with significantly lower angiotensin II (ANG II) but not aldosterone concentrations. In perspective, this feed‐forward Na^+^‐driven GLP‐1/ANGII response could have an impact on renal Na^+^‐handling.

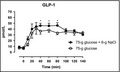

## 1 INTRODUCTION

Hypertension is one of the most common and important health problems worldwide because of its high frequency and concomitant risks of cardiovascular and kidney disease (Kearney et al., 2005; Mills et al., 2016). It has been estimated that about 30% of the world's adult population will have hypertension by the year 2025 (Kearney et al., 2005; Mills et al., 2016). Hypertension is caused by genetic and epigenetic predisposition and modified by environmental influences and sedentary lifestyles. There is overwhelming evidence that high dietary sodium intake increases the risk for incident hypertension and leads to worse cardiovascular outcomes in established hypertension (Farquhar et al., 2015; Whelton et al., 2012). While the kidneys regulate sodium balance and blood pressure, the gastrointestinal (GI) tract has taste/nutrient sensing receptors and sensors for electrolytes (e.g. sodium, potassium, phosphate) partly coupled to release of GI hormones (Furness et al., 2013). Previous studies support the involvement of a gut‐kidney axis in the excretion of a dietary sodium load mediated by GI hormones, including gastrin and CCK, possibly through interaction with renal dopamine receptors (Chen et al., 2013; Liu & Jose, 2013; Liu et al., 2016). Thus, there is increasing evidence of the importance of the GI tract in blood pressure regulation potentially through feed‐forward effects on renal sodium handling. Recently, we demonstrated a natriuretic effect of another GI hormone, glucagon‐like peptide‐1 (GLP‐1) after extracellular fluid volume expansion. The response was associated with suppression of angiotensin II (ANG II) and independent of renal plasma flow and glomerular filtration rate (Asmar et al., 2015, 2016, 2019). Because GLP‐1 release is physiologically regulated by luminal stimuli in the small intestine, and since salt intake varies strongly within and between individuals, a direct salt sensitivity of L‐cell GLP‐1 release could provide a sequential, feed‐forward signal that adds to the gastric/duodenal signals from gastrin/CCK for renal sodium excretion. Whether the L‐cell has sensors for sodium coupled to release of GLP‐1 is not clarified. Specifically, this randomized, controlled cross‐over study was designed to investigate the hypothesis that postprandial GLP‐1 plasma concentrations are sensitive to an increased NaCl intake, supporting a feed‐forward natriuretic system.

## 2 MATERIALS AND METHODS

### 2.1 Design

**Figure 1:**
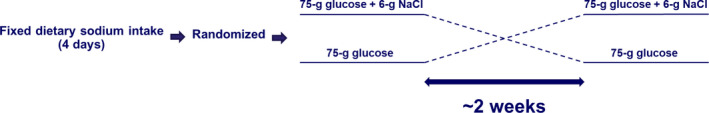
Timeline and the study design. The study was an observational, open‐label, cross‐over, dietary intervention with standard oral glucose load with and without 6 g of NaCl

The study protocol had a randomized, cross‐over design with a washout period of ~2 weeks. Each subject served as his own control and was studied on two different occasions during an oral load of 75 grams of glucose + 6 grams of sodium chloride (NaCl) or a 75 grams of glucose load alone (Figure 1).

### 2.2 Participants

Baseline characteristics are shown in Table 1. Eleven young male participants completed the study. All participants were healthy and none took medication at the time of the study. Body composition was determined by dual energy X‐ray absorptiometry (DXA) scanning (Lunar iDXA; GE Healthcare, Brøndby, Denmark) (Table 1). Consent to participate was obtained after the participants had read a description of the experimental protocol, which was approved by the Scientific Ethics Committee of the Capital region of Copenhagen (H‐17030187). One participant was excluded from the study due to a pathological oral glucose tolerance test. This study was not registered on clinicaltrials.gov, because it was considered as a kinetic observational study with oral ingestion of normal dietary components (salt and sugar) and pure observational measurements.

**Table 1:** – Baseline characteristics of participants

Variable	Value
Number of participants	10
Age (year)	25 ± 4
Height (cm)	179 ± 4
Weight (kg)	72.8 ± 7.7
Lean body mass (kg)	58.4 ± 4.2
Whole body fat mass (kg)	14.4 ± 5.8
Systolic blood pressure (mm Hg)	119 ± 12
Diastolic blood pressure (mm Hg)	75 ± 6
Heart rate (bpm)	72 ± 8
Fasting glucose concentration (mmol/L)	5.1 ± 0.1
Fasting insulin concentration (pmol/L)	74 ± 14

Note

Body composition was determined by dual energy X‐ray absorptiometry (DXA) scanning. Data are presented as means ± *SD*.

### 2.3 Protocol

For 4 days before each experiment, all participants consumed a controlled mixed diet (2,822 kcal per day; 16% protein, 55% carbohydrate, 29% fat) (Figure 1). The food was handed out frozen, and the sodium chloride (NaCl) content of the diet, measured at Eurofins Stein's Laboratory in Denmark, was 55–75 mmol per day. NaCl was added to the diet in order to standardize daily intake at 2 mmol NaCl per kg body weight per day. Twenty‐four‐hour urine was collected on the last day, and electrolytes, glucose, creatinine, and albumin concentrations were determined. Water intake was ad libitum, and strenuous physical activity was not allowed during the 4‐day period.

Participants fasted for 12 hr before the beginning of the experiments around 9 a.m. After emptying the bladder, confirmed by ultrasound, participants remained supine throughout the experiments. A radial artery was catheterized with a 20‐gauge catheter (BD Arterial Cannula®: 1.1 mm OD, length 45 mm, Becton Dickinson, Franklin Lakes, New Jersey, USA) for blood sampling. After approximately 45 min of rest in supine position, two baseline blood samples were drawn followed by an oral load of 75 grams of glucose (416 mmol) + 6 grams of NaCl (103 mmol) or a 75 grams of glucose load alone; both were dissolved in 500 mL tap water. The solutions were prepared freshly and ingested within 5 min. Six grams of NaCl corresponding to 103 mmol was chosen, because it corresponded to the high‐normal content of a major western diet meal.

### 2.4 Blood and urine analyses

Blood was collected from the radial artery throughout the experiments (0–140 min) every 10 min for the first 40 min and thereafter every 20 min for the remaining experiment. The amount of total collected blood (~165 mL) on each study day was carefully substituted with a similar amount of isotonic saline during the experiments.

Arterial blood samples were analyzed for GLP‐1, GIP, CCK, gastrin, insulin, C‐peptide, renin, ANG II, aldosterone, pro atrial natriuretic peptide (ANP), ANP, brain natriuretic peptide (BNP), glucose, sodium, chloride, hydrogen, potassium, hematocrit, and oxygen saturation.

Plasma samples were assayed for total GLP‐1 and GIP immunoreactivity as previously described (Lindgren et al., 2011; Orskov et al., 1994). Gastrin concentrations in plasma were measured, using a radioimmunoassay (RIA) that is specific without cross reactivity with any CCK peptide (Stadil & Rehfeld, 1973), and CCK concentrations in plasma were measured with a specific RIA without cross reactivity with any gastrin peptide (Rehfeld, 1998).

Plasma renin concentrations were measured by RIA of angiotensin I (ANG I) through the antibody‐trapping method of Poulsen and Jørgensen (Poulsen & Jorgensen, 1974), using ethylenediaminetetraacetic acid (EDTA) plasma (100 μL) incubated with plasma from a nephrectomized sheep for 3 hr as previously described. Concentrations were measured by the rate of ANG I formation and standardized in terms of international units per liter (IU/L) according to the activity of the WHO International Standard (ref. no. 68–356; National Institute for Biological Standards and Control, Hertfordshire, UK) of which samples of 0.05 IU/L were included in every run of the renin assay. In the period where measurements were performed, 1 IU of the WHO standard corresponded to 32 ± 5 ng ANG I per hr. Between‐assay coefficient of variation (CV) was 15%. ANG II peptide hormone concentrations in plasma were measured by RIA (using specific antibodies and charcoal plasma to separate bound antigen from free) after extraction performed by use of Sep‐Pak C18 columns (Waters, Millipore Corporation, Milford, MA, USA) based on antibody Ab‐5–030682 raised in rabbits (Bie & Sandgaard, 2000). Briefly, plasma was incubated with antibody (final dilution of 1:1,000,000) and tracer ^125^I‐labeled ANG II (Department of Clinical Physiology, Nuclear Medicine and PET, Rigshospitalet, University Hospital of Copenhagen, Denmark). Blood samples for ANG II analyses were collected in EDTA tubes prepared with 150 μL of 1, 10‐Phenantrolin Monohydrate. Plasma aldosterone concentrations were measured, using a commercial ELISA (MS E‐5200, Labor Diagnostika Nord GmbH & Co. KG, Germany). EDTA plasma was incubated with Aldosterone HRP conjugate for 1 hr as described by the manufacturer and was subjected to the standard procedure in the instructions. A human EDTA plasma pool was used as an internal standard (79 ± 8 pg/mL). Between‐assay coefficient of variation was 10%. Plasma proANP, ANP and BNP were measured by ELISA kits (CUSABIO, Baltimore, USA). The intraassay CV was < 8% for each peptide measured, and the interassay CV was < 4% for proANP and < 10% for ANP and BNP. Blood samples for proANP, ANP, and BNP analyses were collected in EDTA tubes prepared with 62.5 μL of 1,10‐Phenantrolin Monohydrate.

Insulin and C‐peptide levels in plasma were measured, using commercial ELISA (Insulin Human ELISA EIA‐2935 and C‐peptide ELISA EIA‐1293, AH Diagnostics, Aarhus, Denmark).

Plasma electrolyte (Na^+^, Cl^‐^, H^+^, K^+^) concentrations, glucose, hematocrit, and oxygen saturation were measured, using an automated benchtop blood analyzer system (ABL 700 series, Radiometer Medical Aps, Brønshøj, Denmark).

Urinary electrolyte (Na^+^, K^+^) concentrations were measured by atomic absorption (Atomic absorption spectrophotometer model 2,380, PerkinElmer, Norwalk, Connecticut). Urinary pH was measured, using a XC161 Combination pH electrode (Radiometer Medical Aps).

### 2.5 Calculations

Twenty‐four‐hour urinary excretions were calculated from the actual collection time and presented on a 24‐hr basis as follows: electrolyte excretion (V_x_) = U_x_ ∙ V_u_, where U_x_ is the concentration of substance X in urine, and V_u_ is the 24‐hr urine volume.

### 2.6 Statistical analysis

The primary endpoint in this study was the postprandial GLP‐1 plasma concentration response. When we used a two‐tailed α = 0.05 and an 80% power threshold, a sample size of *n* = 9 was calculated to be needed to detect an appreciable effect of a NaCl load in addition to a glucose load on postprandial circulatory levels of GLP‐1. Data were analyzed, using GraphPad Prism 8 (GraphPad Software Inc. La Jolla, CA, USA). Area under the curve (AUC) was calculated using the trapezoidal rule, and the *t*‐test (2‐tailed) for paired data was used for comparing incremental AUC values between the two study days. Values of *p* < .05 were considered statistically significant. We confirmed that data on hormone and electrolyte levels in this study passed the *Shapiro–Wilk test* (GraphPad Software Inc.) for normal distribution.

## 3 RESULTS

### 3.1 Standardized sodium chloride intake

All participants completed the mixed controlled diet with fixed NaCl intake for 4 days prior to each experiment, and all 24‐hr urine collections were successfully completed with similar mean collection time on each study day (23.8 ± 0.6 hr versus 23.3 ± 0.3 hr, *p* = .575). On the last day of the 4‐day period with standardized NaCl intake and just before each study day, 24‐hr urine data were statistically similar, and mean urinary sodium excretion was not different from the calculated expected amount ~ 140 mmol (Table 2).

**Table 2:** – 24‐hr urinary excretions during baseline

Urine variable	Baseline (glucose + NaCl)	Baseline (glucose)	*p*‐value
Number of participants	10	10	‐‐‐
Volume (mL/24‐hr)	1,560 ± 200	1,628 ± 212	.451
Sodium (mmol/24‐hr)	133 ± 11	132 ± 11	.879
pH	5.9 ± 0.1	5.8 ± 0.1	.439
Potassium (mmol/24‐hr)	60 ± 6	59 ± 6	.986

Note

Twenty‐four‐hour urinary excretions were calculated from the actual collection time (23.8 ± 0.6 hr versus 23.3 ± 0.3 hr, *p* = .575) and presented on a 24‐hr basis. Data are presented as means ± SE.

### 3.2 Effect of glucose and sodium chloride intake on arterial plasma concentrations of glucose, insulin, and C‐peptide

**Figure 2:**
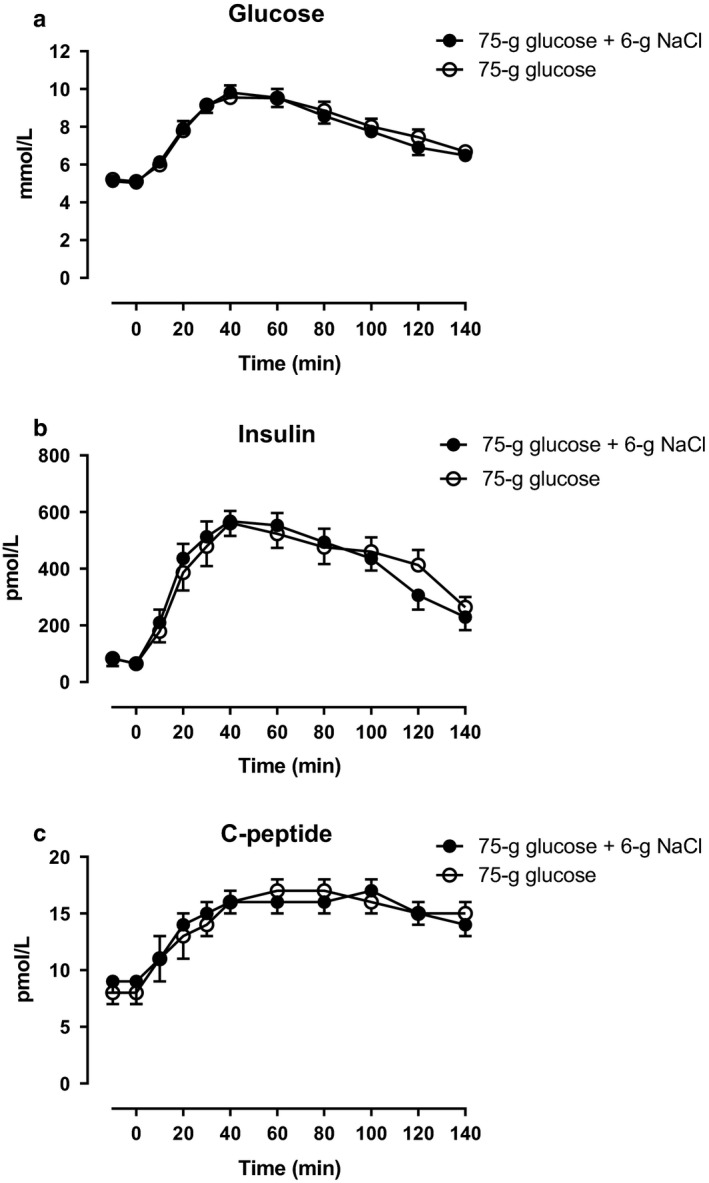
Arterial plasma concentrations of (a) glucose, (b) insulin, and (c) C‐peptide after a 75‐gram oral glucose load (75 g of glucose) with a 6‐gram oral sodium chloride load (6 g of NaCl) (filled circles) or 75 g of glucose alone (open circles) from 0 to 140 min. Data are presented as means ± SE

None of the participants complained about abdominal discomfort specifically during the NaCl load. Arterial plasma concentrations of glucose, insulin, and C‐peptide increased similarly during glucose + NaCl and glucose alone (Figure 2a‐c).

### 3.3 Effect of glucose and sodium chloride intake on arterial plasma concentrations of sodium, chloride, potassium, hydrogen, calcium, and hematocrit

**Figure 3:**
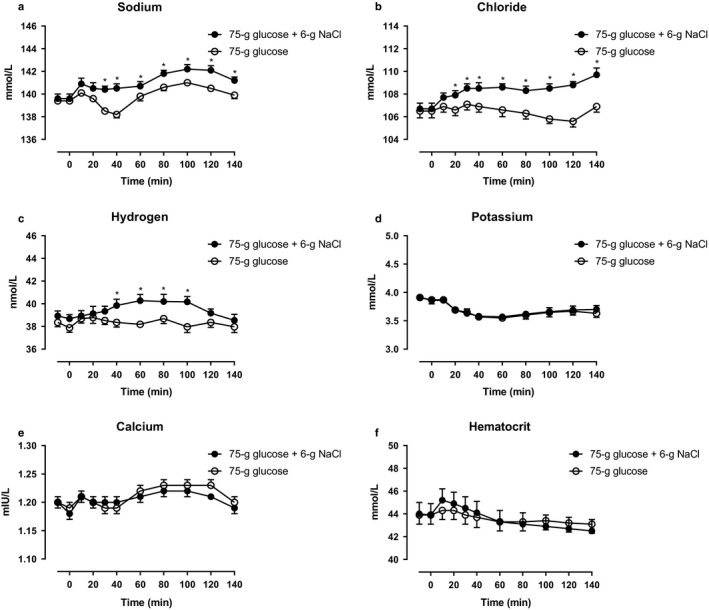
Arterial plasma concentrations of (a) sodium, (b) chloride, (c) hydrogen, (d) potassium, (e) calcium, and (f) hematocrit after a 75‐gram oral glucose load (75 g of glucose) with a 6‐gram oral sodium chloride load (6 g of NaCl) (filled circles) or 75 g of glucose alone (open circles) from 0 to 140 min. *, statistically significant differences (*p* < .05) between incremental integrated concentrations. Data are presented as means ± SE

Arterial plasma concentrations of sodium increased during glucose + NaCl and glucose alone compared to baseline, however, the increase was significantly higher during glucose + NaCl compared with glucose (Figure 3a). Plasma concentrations of chloride and hydrogen only increased significantly during glucose + NaCl (Figure 3b and c). Plasma potassium concentrations decreased similarly during during glucose + NaCl and glucose alone (Figure 3d). Plasma concentrations of calcium, and hematocrit remained unchanged during glucose + NaCl compared with glucose alone (Figure 3e and f).

### 3.4 Effect of glucose and sodium chloride intake on arterial plasma concentrations of GLP‐1, GIP, CCK, and gastrin

**Figure 4:**
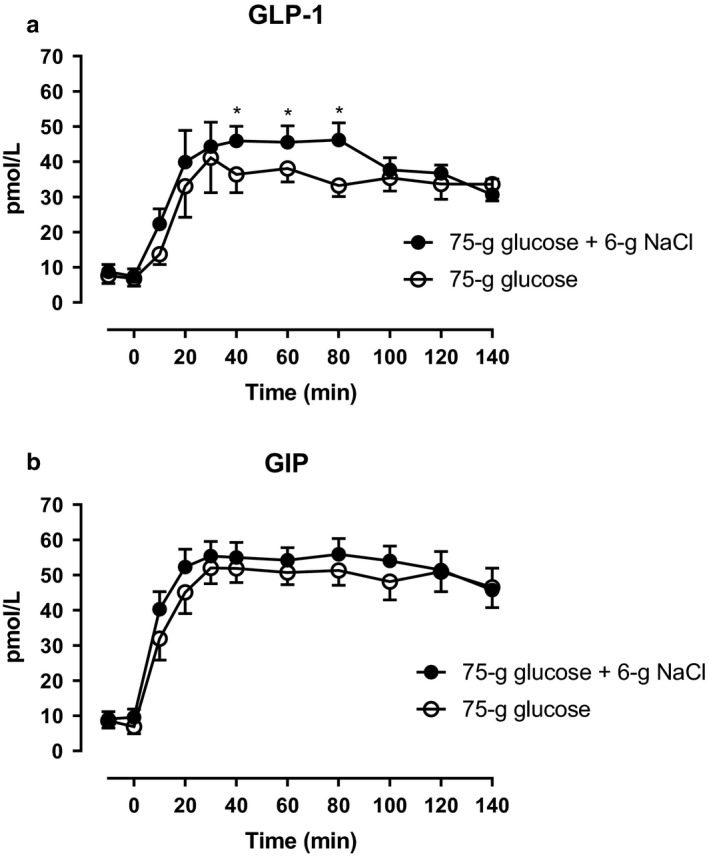
Arterial plasma concentrations of (a) GLP‐1 and (b) GIP after a 75‐gram oral glucose load (75 g of glucose) with a 6‐gram oral sodium chloride load (6 g of NaCl) (filled circles) or 75 g of glucose alone (open circles) from 0 to 140 min. *, statistically significant differences (*p* < .05) between steady state incremental integrated concentrations. Data are presented as means ± SE

**Figure 5:**
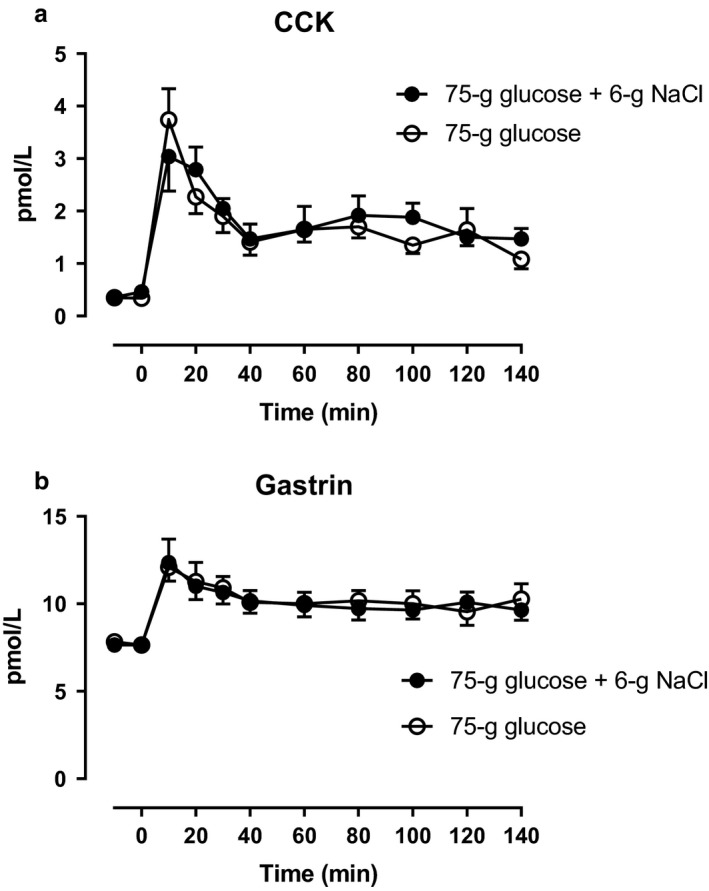
Arterial plasma concentrations of (a) CCK and (b) gastrin after a 75‐gram oral glucose load (75 g of glucose) with a 6‐gram oral sodium chloride load (6 g of NaCl) (filled circles) or 75 g of glucose alone (open circles) from 0 to 140 min. Data are presented as means ± SE

Arterial plasma concentrations of total GLP‐1 increased during glucose + NaCl and glucose alone, however, an apparent plateau (incremental AUC, lasting from 40–80 min) was significantly higher during glucose + NaCl compared with glucose alone (Figure 4a). Arterial plasma concentrations of GIP increased on both days and no statistically significant differences between the days were observed (Figure 4b). Arterial plasma concentrations of CCK and gastrin increased on both days and no statistically significant differences between the days were observed (Figure 5a and b).

### 3.5 Effect of glucose and sodium chloride intake on the renin‐angiotensin‐aldosterone system and natriuretic peptides

**Figure 6:**
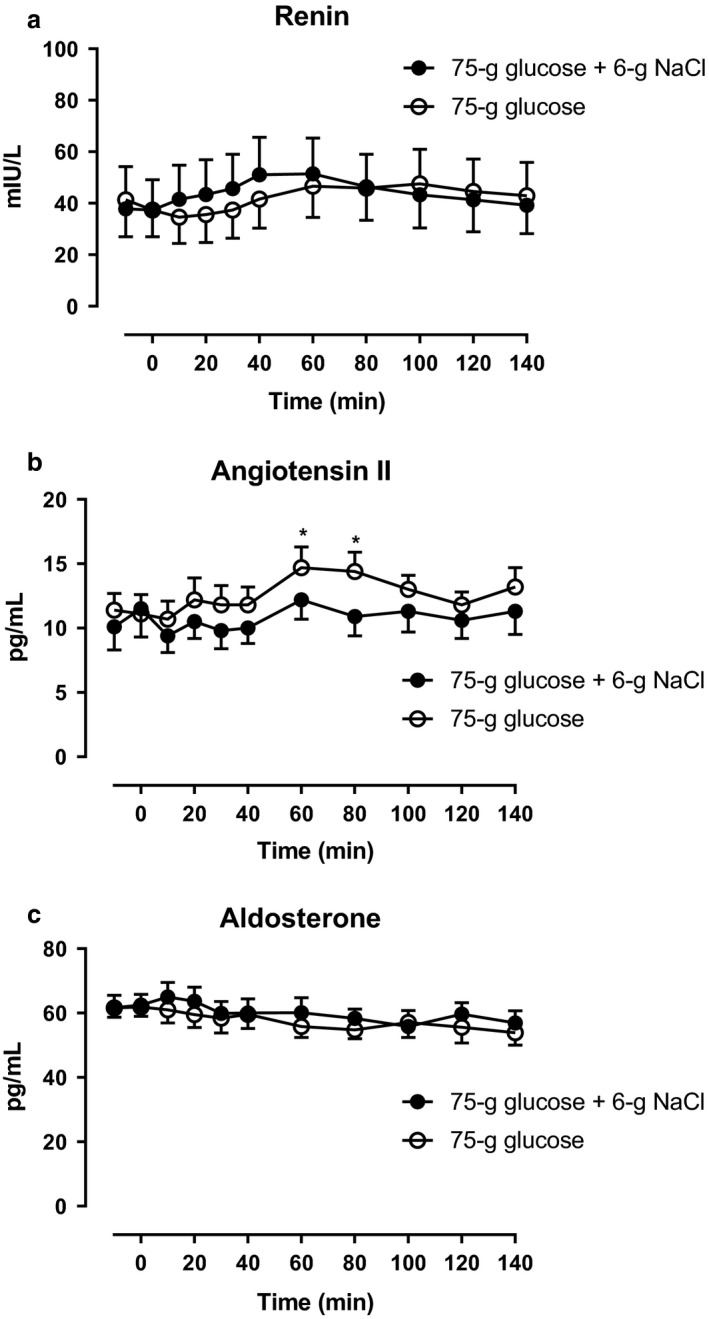
Arterial plasma concentrations of (a) renin, (b) angiotensin II, and (c) aldosterone after a 75‐gram oral glucose load (75 g of glucose) with a 6‐gram oral sodium chloride load (6 g of NaCl) (filled circles) or 75 g of glucose alone (open circles) from 0 to 140 min. *, statistically significant differences (*p* < .05) between steady state incremental integrated concentrations. Data are presented as means ± SE

**Figure 7:**
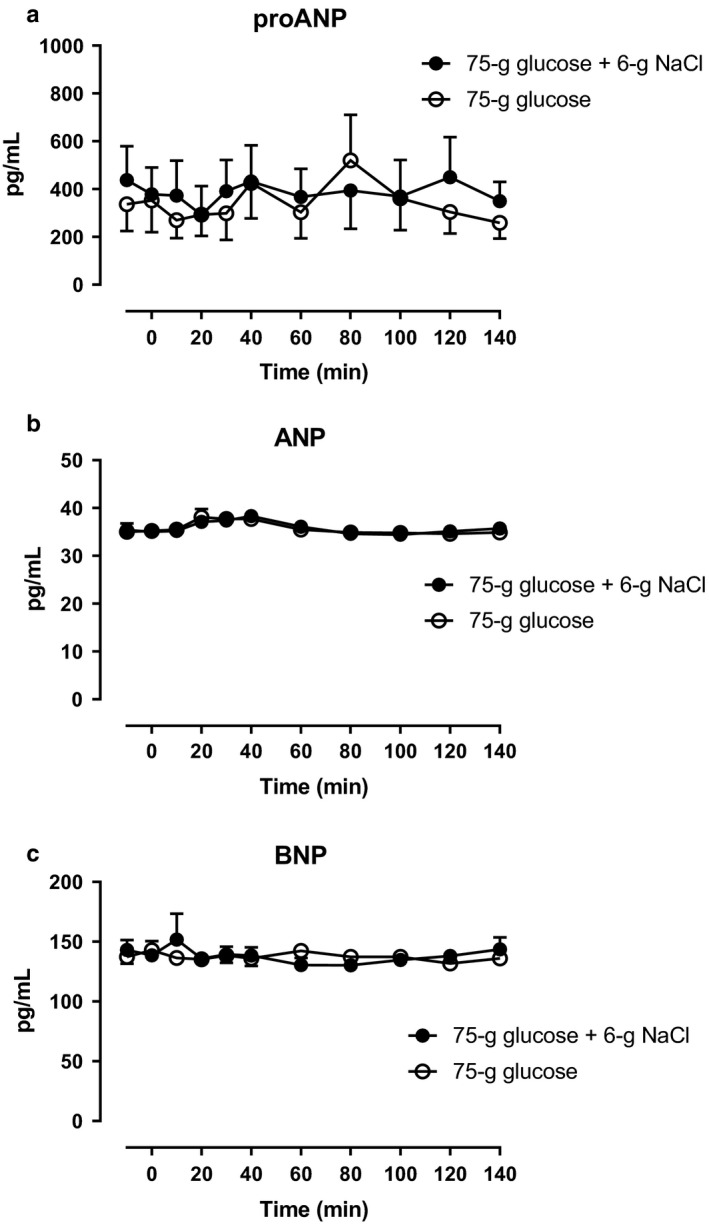
Arterial plasma concentrations of (a) proANP, (b) ANP, and (c) BNP after a 75‐gram oral glucose load (75 g of glucose) with 6‐gram oral sodium chloride load (6 g of NaCl) (filled circles) or 75 g of glucose alone (open circles) from 0 to 140 min. Data are presented as means ± SE

Arterial plasma concentrations of ANG II were significantly lower from 60–80 min (incremental AUC) during glucose + NaCl compared with glucose alone (Figure 6b). Arterial plasma concentrations of renin and aldosterone remained unchanged on both days and no significant differences between the days were observed (Figure 6a and c, respectively). Arterial plasma concentrations of proANP, ANP, and BNP remained unchanged on both days and no statistically significant differences between the days were observed (Figure 7a‐c).

## 4 DISCUSSION

This study supports the hypothesis, that postprandial physiological GLP‐1 responses in humans were enhanced selectively by increased dietary sodium chloride (NaCl) intake when compared with other nutrient‐sensitive GI hormones. GLP‐1 secretion from the small intestine normally increases following nutrient intake, and dietary carbohydrates are major determinants of GLP‐1 secretion (Gribble et al., 2003; Reimann & Gribble, 2002). There is evidence, that the main mechanism behind this is glucose uptake in the enteroendocrine L‐cells mediated via sodium‐glucose cotransporter 1 (SGLT1) (Gribble et al., 2003; Reimann & Gribble, 2002). Under physiological circumstances it would not be expected that sodium availability is rate‐limiting for glucose absorption and thereby GLP‐1 secretion (because of the high abundance of sodium in the diet and the bile and gastropancreatic secretions). Addition of NaCl to the meal increases the osmolarity of the meal, which could be a contributory factor to the increased GLP‐1 secretion mediated by the electrogenic nature of Na^+^‐coupled glucose uptake by SGLT1, highly expressed in L‐cells (Gribble et al., 2003). Thus, SGLT1 is an L‐cell glucose sensor, utilizing the inwardly directed sodium gradient to drive glucose influx which stimulates GLP‐1 secretion to the circulation. Our results indicate that SGLT1 may indirectly act as an L‐cell sodium sensor in which increased dietary, and thus luminal sodium, amplifies glucose/sodium‐dependent GLP‐1 secretion. This is supported by the present study in which arterial levels of sodium increased during both study days, however, the increase was significantly larger during an oral glucose + NaCl load compared with glucose load alone, whereas chloride levels only increased during glucose + NaCl and remained unchanged during glucose alone. Thus, these different effects on plasma sodium and chloride levels most likely reflect the NaCl loading by ~ 100 mmol. From this study, we cannot determine whether the same feed‐forward GLP‐1 secretion was seen if the same amount of NaCl was given but at a concentration at, or lower than, that of plasma. It remains unclarified whether plasma sodium concentrations need to rise in order to be sensed or is it solely driven by a luminal sensing of ingested sodium, e.g. an acute splanchnic input sensor/monitor for sodium intake. However, in healthy volunteers, intraduodenal hyperosmolar saline (1,500 mOsm/L) increased circulatory GLP‐1 levels (physiological plasma levels), peaking around 60 min compared with an isoosmolar solution (300 mOsm/L) (Veedfald et al., 2018).

The increased GLP‐1 plasma concentrations observed during NaCl loading is consistent with the hypothesis that there is a GLP‐1‐mediated feed‐forward natriuretic effect since it was associated with lower circulating plasma levels of ANG II, a major determinant of renal sodium excretion. The significantly lower ANG II concentrations were independent of renin, aldosterone, and proANP/ANP/BNP levels as well as glucose and insulin levels. This is in line with previous human studies (Asmar et al., 2019; Skov et al., 2013) that demonstrated a significant natriuretic effect during exogenous GLP‐1 infusions – reaching physiological levels in 2–3‐hr periods – when the extracellular fluid volume (ECV) was expanded by intravenous sodium loading. In these studies, there was a suppression of ANG II with no change in renal plasma flow and glomerular filtration rate, pointing to a tubular mechanism for reduced NaCl reabsorption secondary to the ANG II suppression. The mechanism by which GLP‐1 reduces ANG II has not been shown. One explanation may be a direct inhibition of ACE‐mediated ANG II synthesis in tissues (Asmar et al., 2019; Skov et al., 2013).

In other studies of healthy participants (Asmar et al., 2015) and patients with type 2 diabetes (Asmar et al., 2016) without intravenous ECV expansion, we were unable to demonstrate a GLP‐1‐induced natriuretic effect. The effect is thus associated with the physiological need to excrete excess sodium and mild expansion of ECV to an extent that does not alter natriuretic peptides. This is in line with previous human studies (Preston et al., 2012) demonstrating that ECV expansion is important in eliciting a rapid‐acting, feed‐forward natriuretic mechanism.

Together, the observations suggest the existence of a causal relationship that needs to be verified by direct experiments. The luminal sensing of ingested sodium suggests the presence of a splanchnic input sensor/monitor for sodium which modulates renal sodium excretion.

Several other gut‐derived hormones have been proposed to influence renal function and electrolyte balance as reviewed comprehensively (Jose et al., 2016; Michell et al., 2008; Muskiet et al., 2017; Thomas & Kumar, 2008; Yang et al., 2017). Thus, animal and human models have shown that supraphysiological infusions of both gastrin and CCK may induce natriuresis (Calam et al., 1987; Chen et al., 2013; Ladines et al., 2001) associated with decreased sodium–hydrogen antiporter 3 activity (Liu & Jose, 2013; Liu et al., 2016) and decreased plasma renin activity (Calam et al., 1987). Furthermore, at least in rats, gastrin may be released from the upper intestine into the circulation in response to sodium intake, and subsequent activation of renal gastrin receptors may act synergistically with renal dopamine receptors to increase sodium excretion (Chen et al., 2013).

Recently, it was demonstrated that a hyperosmolar load alone increases CCK secretion in humans (Veedfald et al., 2018). However, the present observation of similar responses to NaCl intake with glucose compared to glucose alone in humans makes it less likely that CCK is the signal that promotes the additional renal sodium excretion. Thus, the present data show that CCK and gastrin are not likely sensors (or effectors) of an (additional) oral NaCl load in humans under postprandial conditions. In human studies, experiments are needed, generally to investigate causal involvement of ‘intestinal natriuretic peptides’, e.g., uroguanylin as well as guanylin, primarily expressed in the mammalian intestine but also in the kidney (Lorenz et al., 2003; Potthast et al., 2001). Findings in rodents establish a role for uroguanylin in a gut‐kidney axis (Lorenz et al., 2003; Potthast et al., 2001).

## 5 LIMITATIONS OF THE STUDY

In this study we specifically aimed to investigate whether postprandial GLP‐1 secretion compared with the secretion of other nutrient‐sensitive GI hormones is sensitive to an increased NaCl intake. This hypothesis was confirmed as a first step. Whether 10 pmol/L (20%) difference in plasma GLP‐1 between participants ingesting glucose + NaCl versus glucose alone is physiologically relevant, and whether the observed inverse relationship between GLP‐1 and ANG II concentrations is causal in the promotion of urinary sodium excretion was not determined in this observational study and only inferred by ANG II concentrations. Future studies should investigate renal sodium and other electrolyte excretions for longer than 2 hr following similar ingestion protocols and involve blockers of GLP‐1 receptors and/or clamped levels of ANG II and ANG II receptor type 1 (AT1) blockers. Additional studies should be conducted, using oral solutions matched for osmolarity. In this study, participants were given an NaCl loading of ~ 100 mmol in a solution of 200 mmol/L as an add‐on to 75 grams of glucose only during one study day.

Differences in gastric emptying rate should also be considered because of their powerful effects on postprandial hormone and nutrient responses, but this appears less likely since initial plasma responses of all the gut hormones measured as well as glucose absorption profiles were similar between the two study days.

We studied healthy young men, and the more general significance of our findings will need to be tested in a wider range of participants, since studies demonstrate that healthy women have a higher postprandial GLP‐1 response than men (Faerch et al., 2015).

## 6 CONCLUSIONS

In summary, we show that an oral glucose load with added NaCl elicited a larger increase in plasma level of GLP‐1, whereas GIP, gastrin and CCK levels were affected similarly. The larger increase in plasma level of GLP‐1 was associated with lower plasma level of ANG II. It is concluded that dietary sodium content inversely influences postprandial GLP‐1 and ANG II plasma levels.

### 6.1 Perspectives

In perspective, GLP‐1 could potentially support a positive feed‐forward natriuresis that may be secondary to lower ANG II levels. This mechanism could have significant implications for renal sodium handling in health and disease, since there is an impaired incretin secretion and action in obesity and type 2 diabetes. Thus, impaired GLP‐1 sensing may link salt sensitivity with insulin‐resistance, increased sympathetic nervous activity, and ultimately the development of hypertension.

It is interesting that in patients with type 2 diabetes – with high risk of cardiovascular and renal events – treatment with GLP‐1 receptor agonists and to a lesser extent with DPP‐4 inhibitors in addition to standard treatment of diabetes has proven to have beneficial long‐term cardiovascular and renal effects – plausibly beyond the effects of glycemic control (Muskiet et al., 2017). The beneficial effects of the GLP‐1 receptor agonists might represent restoration of the gut‐kidney cross talk.

## CONFLICT OF INTEREST

A.A. and J.J.H. consulted for Novo Nordisk. No conflicts of interest, financial or otherwise, are declared by the authors.

## AUTHOR CONTRIBUTIONS

A.A. designed the experiment, collected the data, performed data analyses and statistics, and wrote the manuscript; P.K.C collected the data and contributed to the manuscript; M.A. participated in designing the experiment, participated in data analyses and statistics, and contributed to the manuscript; L.S. participated in data analyses and statistics and contributed to the manuscript; C.M.S performed the urine analyses and contributed to the manuscript; S.M. contributed to the manuscript; C.M. performed the plasma proANP, ANP, and BNP analyses and contributed to the manuscript; J.J.H. and B.H. performed the plasma GLP‐1 analyses and contributed to the manuscript; J.F.R. performed the CCK and gastrin analyses and contributed to the manuscript; P.H. contributed to the manuscript; B.L.J. performed plasma renin, angiotensin II, and aldosterone analyses and contributed to the manuscript; J.B. participated in designing the experiment, participated in data analyses and statistics, and contributed to the manuscript.

7
